# Vitamin E and antioxidant activity; its role in slow coronary flow

**DOI:** 10.5830/CVJA-2013-076

**Published:** 2013-11

**Authors:** Veysel Kenan Celik, İmge Ezgi Eken, Hüseyin Aydin, Gürsel Yildiz, Mehmet Birhan Yilmaz, Ahmet Gurlek

**Affiliations:** Department of Biochemistry, Faculty of Medicine, Cumhuriyet University, Sivas, Turkey; Department of Biochemistry, Faculty of Medicine, Cumhuriyet University, Sivas, Turkey; Department of Biochemistry, Faculty of Medicine, Cumhuriyet University, Sivas, Turkey; Clinic of Nephrology, Atatürk State Hospital, Zonguldak, Turkey; Department of Cardiology, Faculty of Medicine, Cumhuriyet University, Sivas, Turkey; Department of Cardiology, Faculty of Medicine, Cumhuriyet University, Sivas, Turkey

**Keywords:** vitamin E, antioxidant activity, slow coronary flow

## Abstract

**Aim:**

Oxidative stress, which is widely recognised as an important feature of many diseases, can be defined as an increased formation of reactive oxygen species or decreased antioxidant defense. In this study we measured plasma vitamin E levels and total antioxidant activity (AOA) in patients with slow coronary flow (SCF).

**Methods:**

The plasma vitamin E levels and AOA were measured in 40 patients with angiographically diagnosed SCF. Forty subjects with normal coronary flow (NCF) served as the control group. SCF and NCF were analysed, and blood samples were taken for plasma vitamin E levels and AOA. Plasma vitamin E levels and AOA in patients with SCF were evaluated and compared to those of patients with NCF.

**Results:**

There was no significant difference between the two groups in terms of plasma AOA, lipid profile and C-reactive protein (CRP) levels but there was a significant difference in vitamin E levels between the two groups (*p* = 0.001).

**Conclusion:**

Vitamin E levels were found to be lowered in patients with SCF compared to the NCF group. The association between smoking and vitamin E levels is worth further investigating in larger samples.

## Abstract

The ability of antioxidant defense to scavenge reactive oxygen species (ROS) is important to protect tissues from oxidative damage. Cells and biological fluids have an array of protective antioxidant mechanisms, enzymatic (superoxide dismutase, catalase, glutathione peroxidase) and non-enzymatic, referred to as chain-breaking antioxidants (tocopherols, ubiquinol, carotenoids and flavonoids as lipid phase, and ascorbate, urate, glutathione and other thiols as aqueous phase), both for preventing the production of free radicals and for repairing oxidative damage.^1^-^4^

A free radical contains an unpaired electron in an atomic orbital. In this state, no molecular species is stable for long. A free radical will attract other molecules and either give or receive an electron to make itself thermodynamically stable.^5^ The most important free radicals in many disease states are oxygen derivatives such as hydrogen peroxide, superoxide and particularly, hydroxyl radical, which is the most harmful for tissues.^1^

Transition metals contain one or more unpaired electrons and are therefore also radicals when in the elemental state. However, their key property from the point of view of free radical biology is their variable valency, which allows them to undergo reactions involving the transfer of a single electron. The most important transition metals in human disease are iron and copper.^6^ These elements play a key role in the production of hydroxyl radicals *in vivo*.^7^

Hydrogen peroxide and superoxide can be detoxified enzymatically in the mammalian system by catalase and superoxide dismutase, respectively. However there is no enzymatic system that converts or detoxifies hydroxyl radicals. A hydroxyl radical can be detoxified by non-enzymatic systems. One of these is the tocopherols (α, β, γ and δ), which have a chromanol ring and a phenyl tail, and differ in the number and position of the methyl groups on the ring. The most important lipid-phase antioxidant is probably vitamin E.^8^-^10^

The coronary slow-flow phenomenon was first described in 1972 by Tambe *et al.*^11^ The phenomenon is an angiographic finding characterised by delayed distal vessel opacification in the absence of significant epicardial coronary disease. However, since that time, only a limited number of studies have focused on the aetiology of this unique angiographic phenomenon.

Histopathological studies have revealed the loss of luminary diameter, and capillary and endothelial damage in these patients.^12^ Although the pathophysiological mechanisms of slow coronary flow phenomenon remain uncertain, there are several hypotheses that have been suggested, including endothelial activation and inflammation.^13^ However, the phenomenon is not well studied and deserves further investigation.

In the present study, we investigated plasma vitamin E levels and antioxidant activity in patients with slow coronary flow (SCF) and compared them with those with normal coronary flow (NCF).

## Methods

Forty consecutive patients with angiographically diagnosed SCF in all three epicardial coronary arteries, and 40 subjects with normal coronary flow as a control group were enrolled in our study after obtaining informed consent. All patients underwent selective coronary angiography via the Judkins technique.

Coronary flow of both groups was documented by the thrombolysis in myocardial infarction (TIMI) frame count (TFC). The TIMI frame count method is a simple, reproducible and quantitative index of coronary flow.^14^ To obtain corrected TFC for the left anterior descending (LAD) coronary artery, the TIMI frame count was divided by 1.7. The mean TFC for both groups was calculated by adding the TFC for the LAD, left circumflex artery (LCx) and right coronary artery (RCA), and then dividing the obtained value by three.

The TFC in the LAD and the LCx were assessed in a right anterior oblique projection with caudal angulation (right anterior oblique caudal view) and TFC in the RCA was assessed in a left anterior oblique projection with cranial angulation (left anterior oblique cranial view). All angiograms were filmed at 30 frames/sec.

The TFC, a quantitative method of assessing coronary artery flow, was evaluated on the three main coronary branches (LAD, LCX and RCA), using the protocol described by Gibson *et al.*14 Patients with a corrected TFC greater than two standard deviations from the normal published range (36.2 ± 2.6 frames for LAD, 22.2 ± 1.4 for LCx and 20.4 ± 3 for RCA) for the particular vessel were considered as having slow coronary flow, while those whose corrected TFC fell within two standard deviations (cut-off value for the LAD > 38 frames, for the LCx > 28 frames, for the RCA > 26 frames) of the published normal range were labelled as having normal coronary flow.^14^

Patients with a history of coronary artery disease, recent myocardial infarction or an acute coronary syndrome (within the last two months), coronary vasospasm, coronary ectasia, left ventricular dysfunction, echocardiographically proven left ventricular hypertrophy, uncontrolled hypertension, renal dysfunction and connective tissue disease were excluded from the study. Additionally, patients in both groups who had taken any vitamin supplements within the previous eight weeks were also excluded from the study. All subjects were informed about the study and written consent was obtained from each.

Venous blood samples were collected into tubes containing ethylenediamine tetraacetic acid (EDTA) after an eight-hour fast and immediately stored on ice at 4°C. The plasma was then separated from the cells by centrifugation at 3 000 rpm for 10 min and stored in several aliquots at –80°C until assayed.

Chemicals 2-thiobarbituric acid (TBA), α-tocoferol, and 2.4.6-tris (2-pyridyl)-s-triazine (TPTZ) were supplied by Sigma-Aldrich (Steinheim, Germany). All other chemicals used were obtained from Merck Darmstadt (Germany) and were of analytical grade.

Plasma AOA was measured using a method defined as AOA by Koracevic.^15^ In this method, the hydroxyl radical, the most potent biological radical, is produced in a standardised solution of Fe-EDTA complex reacted with hydrogen peroxide by a Fenton-type reaction. These reactive oxygen species degrade benzoate, resulting in the release of thiobarbituric acid reactive substances (TBARS).^16^,^17^ Antioxidants from the added sample of human fluid cause suppression of the production of TBARS. The inhibition of colour development is defined as AOA.

Plasma vitamin E levels were measured using a spectrophotometric method developed by Martinek.^18^ The assay results are expressed in μmol/l.

## Statistical analysis

Parametric data were expressed as mean ± standard deviation, and categorical data as percentages. SPSS 16.0 (SPSS, Inc, Chicago, Illinois) was used to perform statistical procedures. Continuous variables were tested for normal distribution with the Kolmogorov-Smirnov test. Parametric data were evaluated by independent samples *t*-test, non-parametric data were evaluated by Mann-Whitney *U*-test, and categorical data via chi-square test. A *p*-value ≤ 0.05 was accepted as significant.

## Results

Clinical and laboratory characteristics of the subjects are summarised in Table 1. Since mean TFC was higher in the study group as it was an enrolment criterion, it is not discussed in detail in the text. There were no significant differences in age, gender, hypertension, lipid profile, CRP levels and diabetes, except for smoking, between patients with SCF and controls. There were no significant differences in AOA levels between the two groups. However, plasma vitamin E levels in the SCF group were significantly lower than in the NCF group (Table2).

**Table 1:** Clinical And Laboratory Characteristics Of The Groups

*Variables*	*SCF group (n = 40)*	*NCF group (n = 40)*	p*-value*
Age (year)	51 ± 12	48 ± 10	NS*
Gender: female/male, *n* (%)	13 (32.5)/27 (67.5)	21 (52.5)/19 (47.5)	NS**
Hypertension, *n* (%)	37 (92.5)	25 (62.5)	NS**
Diabetes mellitus, *n* (%)	3 (7.5)	2 (5)	NS**
Smoking, *n* (%)	27 (67.5)	12 (30)	0.001**
Triglycerides (mg/dl)	175 ± 112	169 ± 103	NS*
Total cholesterol (mg/dl)	187 ± 28	179 ± 29	NS*
HDL cholesterol (mg/dl)	38 ± 9	42 ± 15	NS*
LDL cholesterol (mg/dl)	107 ± 24	104 ± 25	NS*
CRP (mg/l)	6 ± 3.1	5.9 ± 2.6	NS*

Data expressed as mean ± standard deviation. *Independent samples *t*-test, **Chi-square test. CRP, C-reactive protein; HDL, high-density lipoprotein; LDL, low-density lipoprotein; NCF, normal coronary flow; NS, not significant; SCF, slow coronary flow.

**Table 2:** Antioxidant Activity And Vitamin E Levels In The Study Groups

*Variables*	*NCF group (n = 40)*	*SCF group (n = 40)*	p-value*
Vitamin E (μmol/l)	109.5 ± 31.6	85.6 ± 28.5	0.001
Antioxidant activity (μmol/l)	2.5 ± 0.6	2.7 ± 0.5	0.139

*Independent samples *t*-test. NCF, normal coronary flow; SCF, slow coronary flow.

Among the non-smokers in both groups, vitamin E levels were lower in those with SCF compared to the control group (Table 3). However, among smokers in both groups, there was no significant difference in vitamin E levels. There was no significant difference in antioxidant activity between the groups and within each group in smokers and non-smokers with normal coronary flow and slow coronary flow (Table 4).

**Table 3:** Vitamin E Levels Within Groups And Between Groups

	*Smokers*		*Non-smokers*		
*Variables*	*n*	*Vitamin E (μmol/l)*	*n*	*Vitamin E (μmol/l)*	p-value*
NCF group	12	95.4 ± 25.4	28	115.5 ± 32.4	0.082
SCF group	27	84.4 ± 28.6	13	88.2 ± 29.2	0.665
*p*-value*		0.248		0.015	

*Mann-Whitney *U*-test

**Table 4:** Antioxidant Activity Levels Within Groups And Between Groups

	*Smokers*		*Non-smokers*		
*Variables*	*n*	*Antioxidant activity (μmol/l)*	*n*	*Antioxidant activity (μmol/l)*	*p*-value*
NCF group	12	2.4 ± 0.5	28	2.7 ± 0.7	0.070
SCF group		2.7 ± 0.6		2.7 ± 0.3	0.938
*p*-value*	27	0. 072	13	0.752	

*Mann-Whitney *U*-test.

## Discussion

Slow coronary flow phenomenon is an important clinical entity because it may be the cause of angina at rest or during exercise, acute myocardial infarction and hypertension.^19^,^20^ There have been several hypotheses suggested for slow coronary flow phenomenon since first described in 1972 by Tambe *et al.*^11^ In this theory, endothelial activation and inflammation, which have been reported to be a major contributing factor to many cardiovascular events and demonstrated to be associated with different clinical settings of coronary artery disease, are the most acceptable hypotheses for SCF.^13^, ^21^-^23^

The biological oxidative effects of free radicals on cells, DNA, proteins and lipids are controlled by a spectrum of exogenous dietary and endogenous antioxidants.^24^,^25^ Oxidative stress occurs when there is an imbalance between free radical production and antioxidant capacity. This may be due to increased free radical generation and/or loss of normal antioxidant defense.

In the vascular wall, decreases in antioxidant defense are thought to alter several important physiological functions. Regulation of blood flow, inhibition of platelet aggregation, inhibition of leukocyte adhesion and control of cellular growth are influenced by oxidant stress. These phenomena ultimately modulate vessel diameter, remodelling and lesion formation.^26^,^27^

Within the lipid interior of membranes, lipophilic radicals are formed that are different from those seen in the intracellular aqueous milieu. Lipophilic radicals require different types of antioxidants such as vitamin E, β-carotene, co-enzyme Q_10_ and membrane structural organisation (phospholipids: cholesterol, different types of phospholipids and fatty acids important for membrane integrity) for their removal.

Vitamin E has a unique biochemical role with both a chainbreaking property and lipoprotein antioxidant. In fact, vitamin E is a poor antioxidant outside the membrane bilayer, but very effective when incorporated into the membrane. Therefore, it can protect cell membranes from oxidative damage and this explains why vitamin E is the most important biological antioxidant but also one of the least important plasma antioxidants.^28^

Oxidative stress has been implicated in over a hundred disorders, including cardiovascular diseases,^1^,^6^,^27^,^29^ but there have been only a few investigations on oxidative stress or antioxidant status in patients with SCF.^23^ Furthermore, to the best of our knowledge, there is no study about vitamin E levels in patients with SCF in the literature.

In the present study, we investigated AOA producing hydroxyl radicals *in vitro* using the Fenton reaction to observe antioxidant defense potential in patients with SCF, and measured vitamin E levels as a component of the antioxidant systems. We observed no difference between the two groups with regard to plasma antioxidant activity, but there was a significant difference in vitamin E levels between the two groups (*p* = 0.001). These results suggest that decreased vitamin E levels as a component of the antioxidant systems can be inadequate to protect endothelial cells from oxidative damage at the tissue level, which is less associated with total plasma activity of hydrophilic compartments.

Concerning the lack of significant difference among smokers in both groups, we have no explanation other than the potentially negative influence of smoking on vitamin E levels. Since we did not evaluate the TIMI frame counts individually, it would have been interesting to see how smoking and its frequency could affect coronary flow in smokers, even if they were within the normal range of TIMI frame counts.

Since we did not include TIMI frame counts individually, our study was limited in terms of correlative association between the coronary flow rate and vitamin E levels. This could be investigated in another study. There may also be other confounding factors that were not considered in the current study. However, given the lack of information on the pathophysiology of slow coronary flow, this can be regarded as an initial study.

The negative influence of smoking on vitamin E levels is interesting. Smoking, in addition to its many hazardous effects on the whole body, appears to render the endothelial membrane weak and exposed to the harmful influences of lipophilic radical attacks by having a negative effect on vitamin E levels.

## Conclusion

Our study has shown that levels of vitamin E, a membrane protector against oxidative stress, were decreased in patients with slow coronary flow. There also appeared to be a clear negative influence of smoking on vitamin E levels. The association between smoking and vitamin E levels is worth further investigation in larger samples.
